# Supplementary material for: Hydraulic-driven adaptable morphing active-cooling elastomer with bioinspired bicontinuous phases
Source: Nat Commun. 2024 Feb 8;15:1179. doi: 10.1038/s41467-024-45562-y (PMC10853206; doi:10.1038/s41467-024-45562-y)
Supplement: Supplementary file 1 — Supplementary Information [file 41467_2024_45562_MOESM1_ESM.pdf]

Supplementary Information

Hydraulic-driven Adaptable Morphing Active-cooling Elastomer with Bioinspired Bicontinuous Phases

Dehai Yu[1,2], Zhonghao Wang[1,2], Guidong Chi[1], Qiubo Zhang[1], Junxian Fu[1], Maolin Li[1], Chuanke Liu[1], Quan Zhou[1], Zhen Li[1], Du Chen[1], Zhenghe Song[1], Zhizhu He[1] ✉

[1]Center for Agricultural Flexible Electronics Technology, College of Engineering, China Agricultural University, Beijing 100083, China.

[2]These authors contributed equally.

✉ Corresponding author. Email: zzhe@cau.edu.cn

This PDF file includes:

Supplementary Figures 1 to 20

Supplementary Note 1

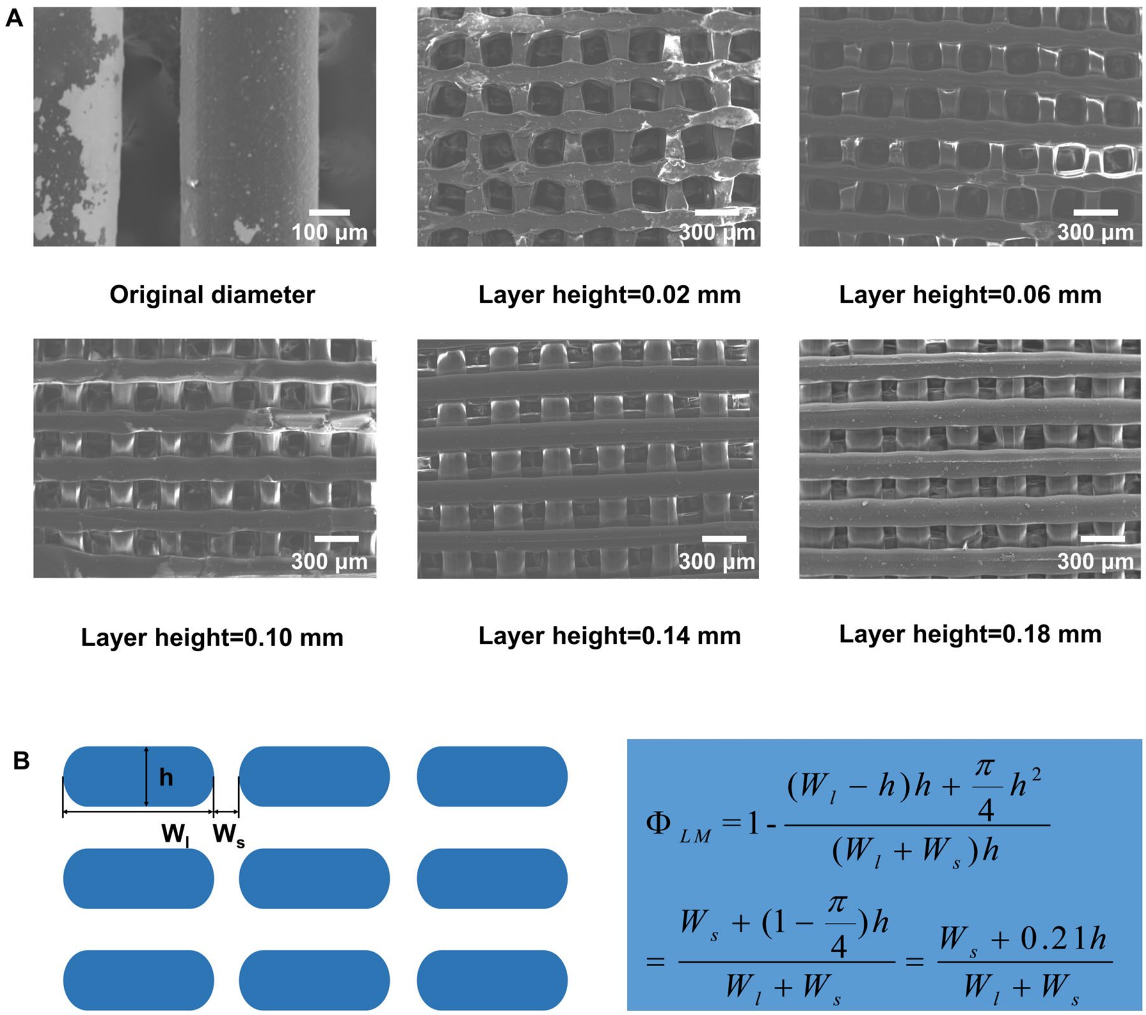

Supplementary Fig. 1. The characterization and schematics of the FDM layer-by-layer printing. **(A)**SEM images of FDM-printed ABS with different commanded layer heights. **(B)** The schematic and computational formula of obtaining the designed porosity and LM perfusion volume ratio.

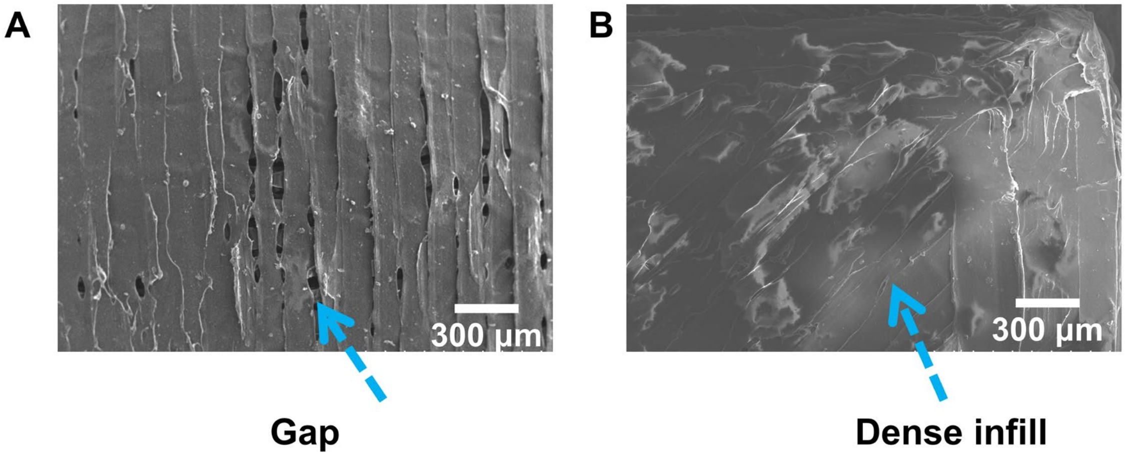

Supplementary Fig. 2. SEM images of FDM-printed ABS. **(A)** FDM-printed ABS existing gap. **(B)** FDM-printed ABS with dense infill designed by optimal printing parameters.

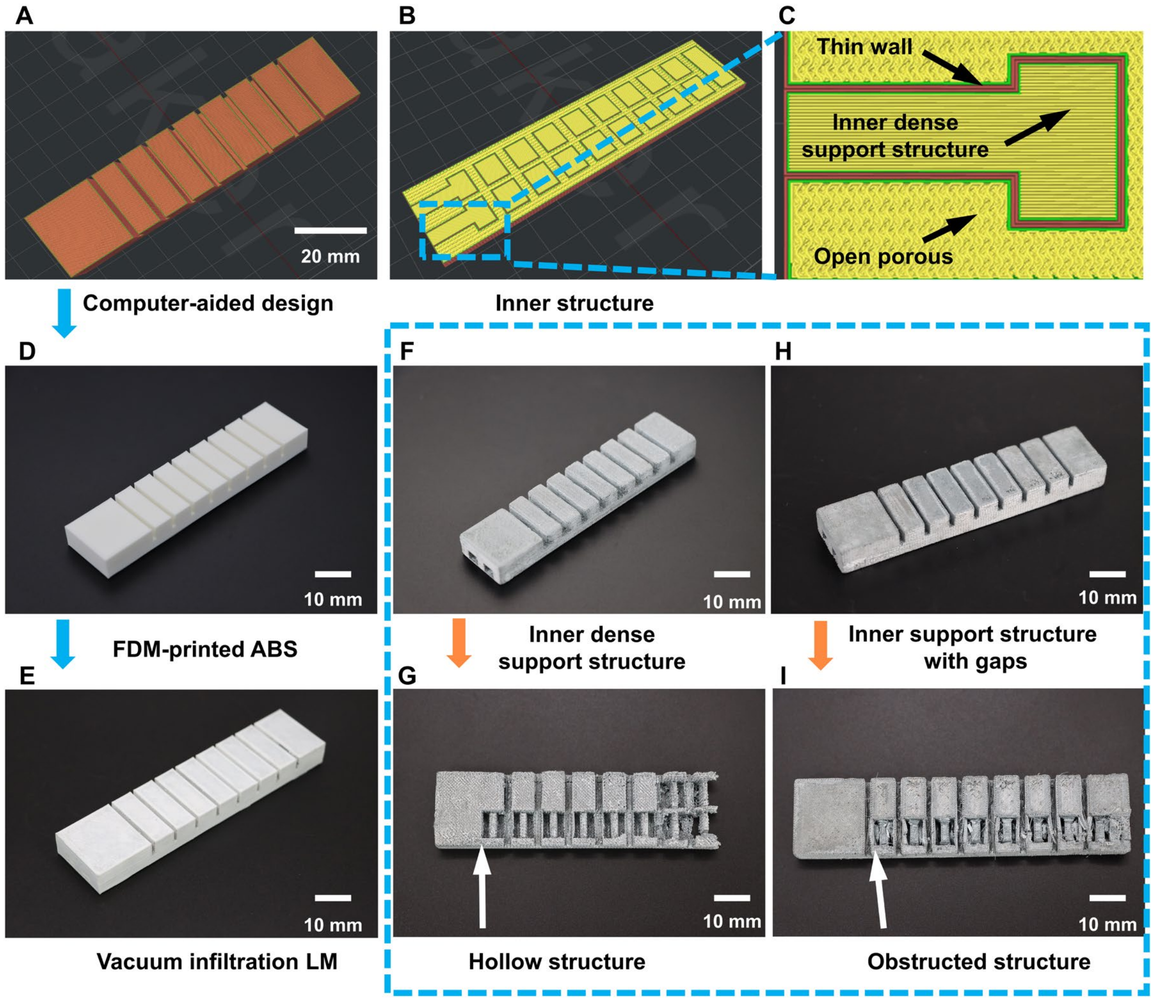

Supplementary Fig. 3. The demonstration and photograph of the tight structure thoroughly prevent LM from perfusing into the support part. **(A)** FDM-printed ABS model with computer-aided design. **(B, C)** The internal cross-section of the designed model. **(D)** Optical photograph of the FDM-printed ABS mold. **(E)** Optical photograph of the mold with vacuuming infiltration LM. **(F, G)** Optical photographs of complicated LMS geometry with hollow structure after the ABS full dissolution with the inner dense support structure. **(H, I)** Optical photographs of complicated LMS geometry with obstructed structure after the ABS full dissolution with the inner support structure with gaps.

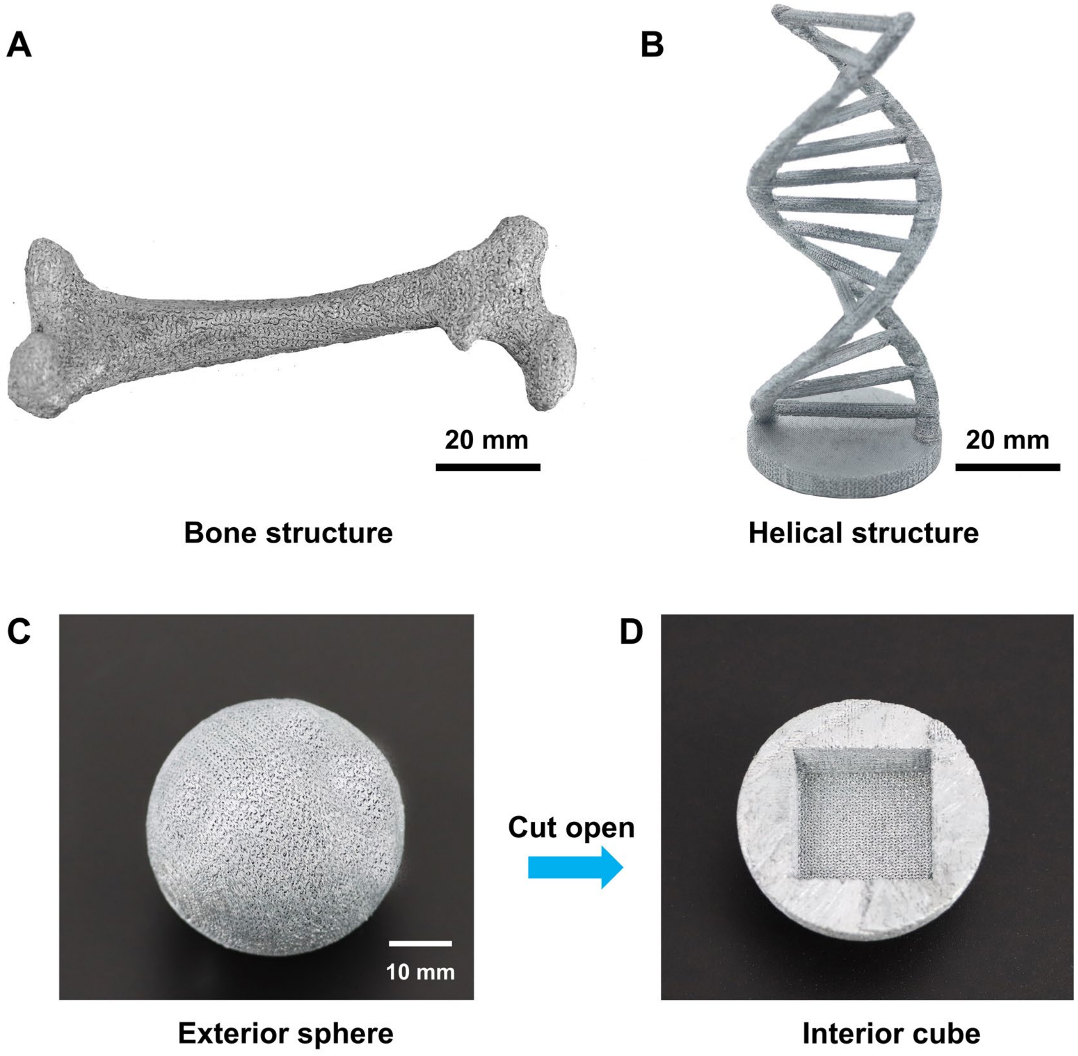

Supplementary Fig. 4. Optical photographs of complicated LMS geometry. **(A)** Bone structure. **(B)** Helical structure. **(C, D)** The structure with inner completely isolated hollow cavities.

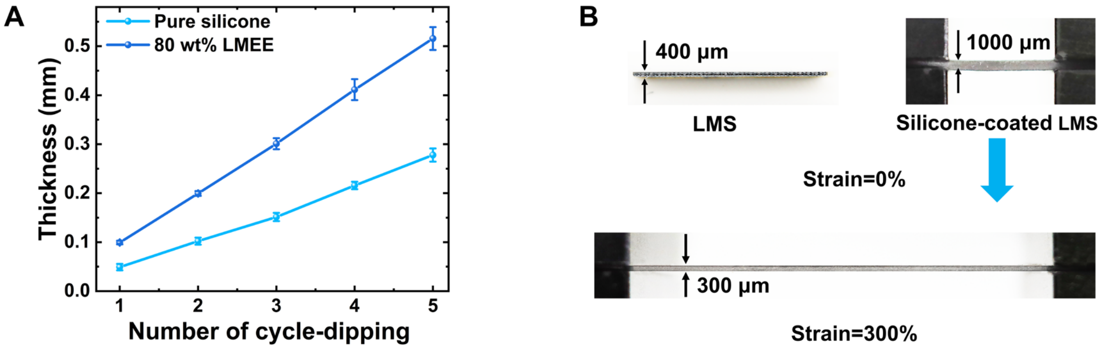

Supplementary Fig. 5. Coating layer thickness of LMS. **(A)** Impacts of the dipping number on the coating layer thickness for the coating materials of pure silicone or LMEE (80 wt% LM). **(B)** LMS (with a thickness of 0.4 mm) is coated with silicone (a thickness of 0. 3mm) to obtain a thickness of 1 mm, which reduces to 0.3 mm (the coating thickness of about 0.09 mm) at a strain of 300 %. Values in **A** represent the mean with error bars (n = 3; independent samples).

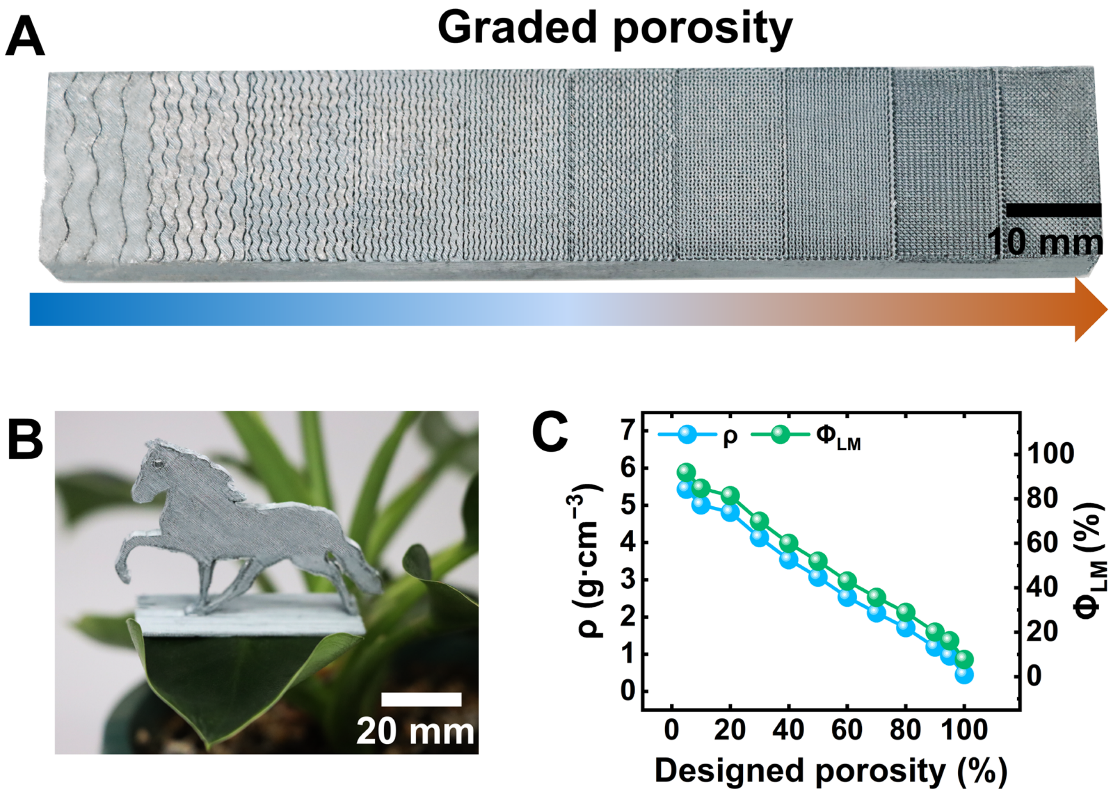

Supplementary Fig. 6. LMS with different porosity. **(A)** LMS with graded porosity. **(B)** Lightweight geometry with an effective density of 0.45 g/cm^3. **(C)** Equivalent density and Φ_{LM} of the LMS versus the designed porosity of ABS model in the slicer software.

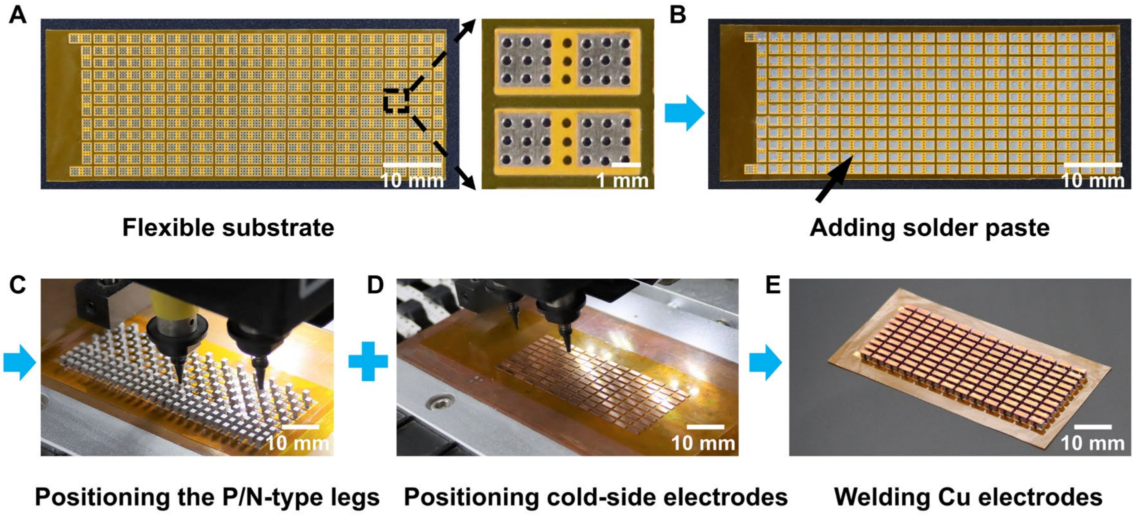

Supplementary Fig. 7. The fabrication process of FTED. **(A)** The photograph of FTED flexible substrate with sandwich structure. **(B)** Adding solder paste to the flexible substrate. **(C)** Positioning the P/N-type legs. **(D)** Positioning cold-side Cu electrodes. **(E)**Welding Cu electrodes onto the P/N-type legs to fabricate FTED.

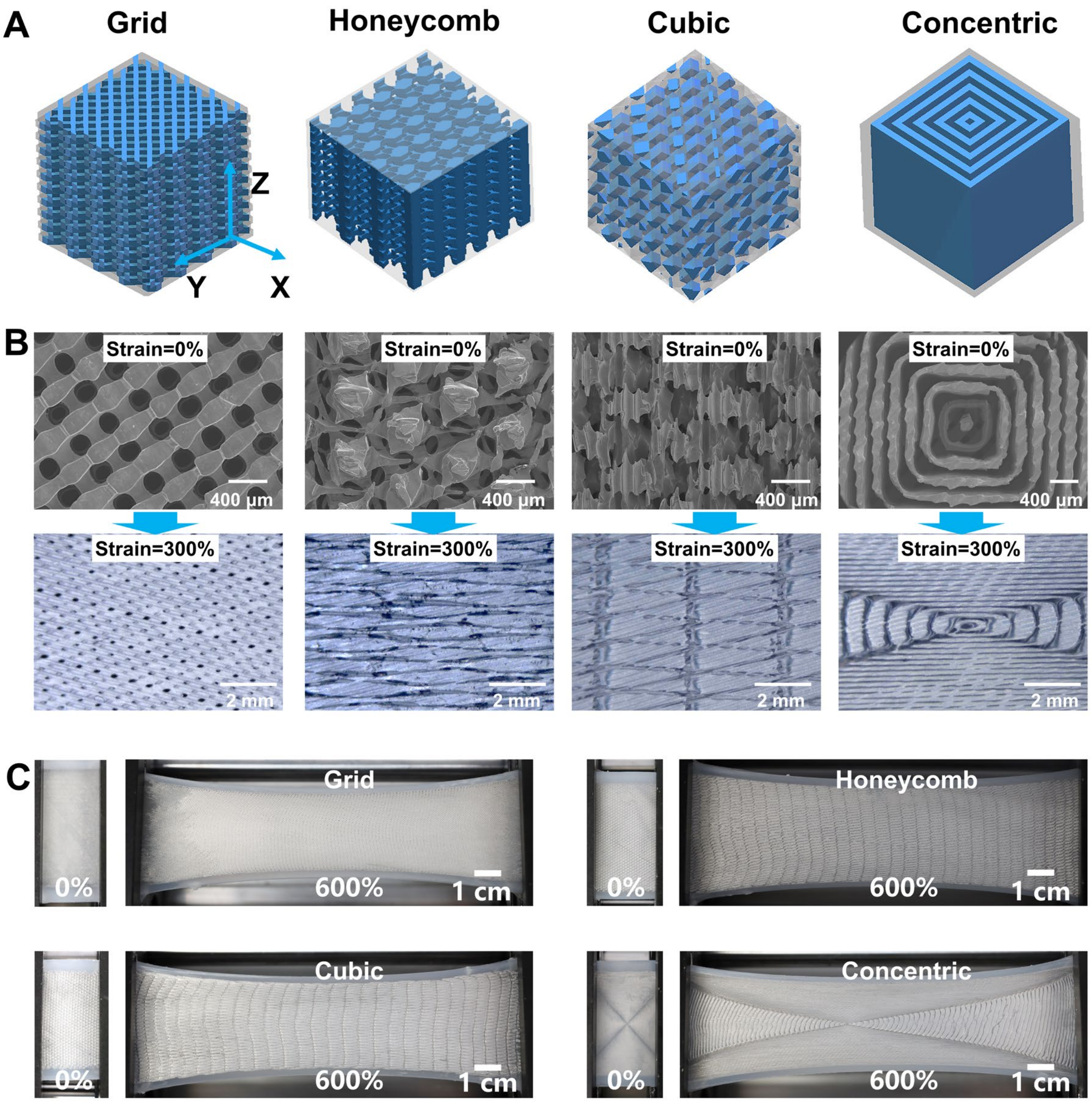

Supplementary Fig. 8. Mechanical characteristics of LMSE. (A) Illustrations of ABS infilling pattern of FDM printing, including types of Grid, Honeycomb, Cubic, Concentric. (B) SEM of LMS micro-structures for different infilling patterns and optical images of LMSE with different LMS patterns under the strain of 300%. **(C)** Optical photographs of the different topological micro-structure of LMSE stretching to 600% strain. Grid, Φ_{LM}=42.9%; Honeycomb, Φ_{LM}=59.3%; Cubic, Φ_{LM}=48.1%; Concentric, Φ_{LM}=44.5%.

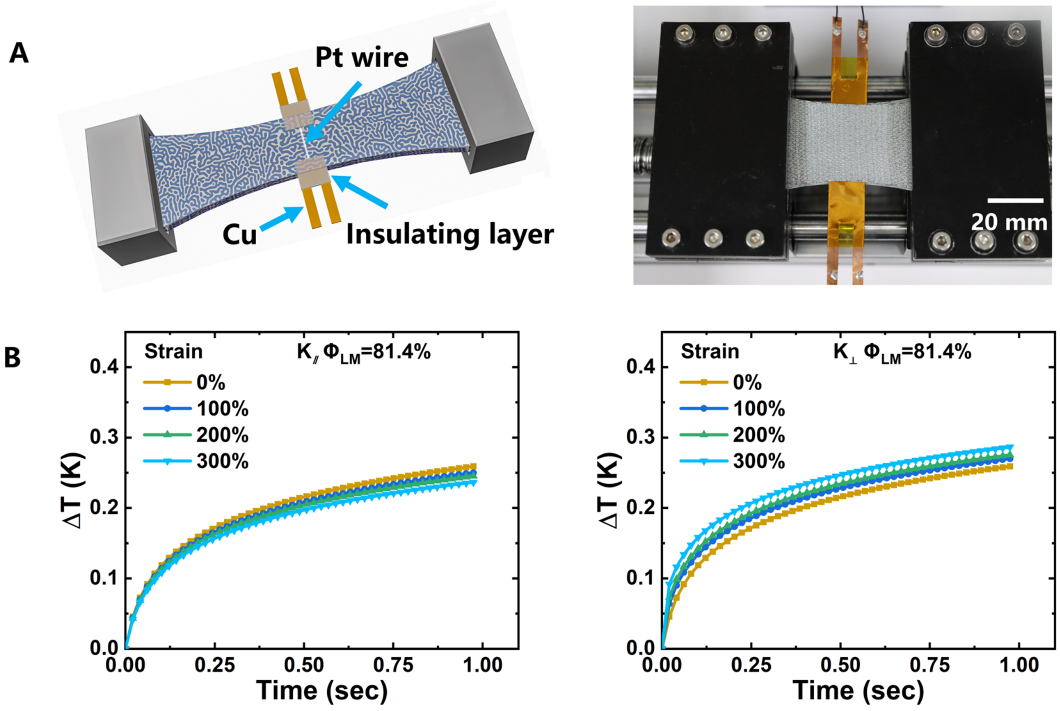

Supplementary Fig. 9. The test platform of LMSE thermal conductivity through a transient hot-wire method. **(A)** Illustration of Pt wire location and the photograph of the test platform. **(B)** Temperature rise versus time with pulse currents for LMSE (Φ_{LM}=81.4%) with transverse-stretching($K_{/\!/}$) and axial-stretching($K_{\perp}$).

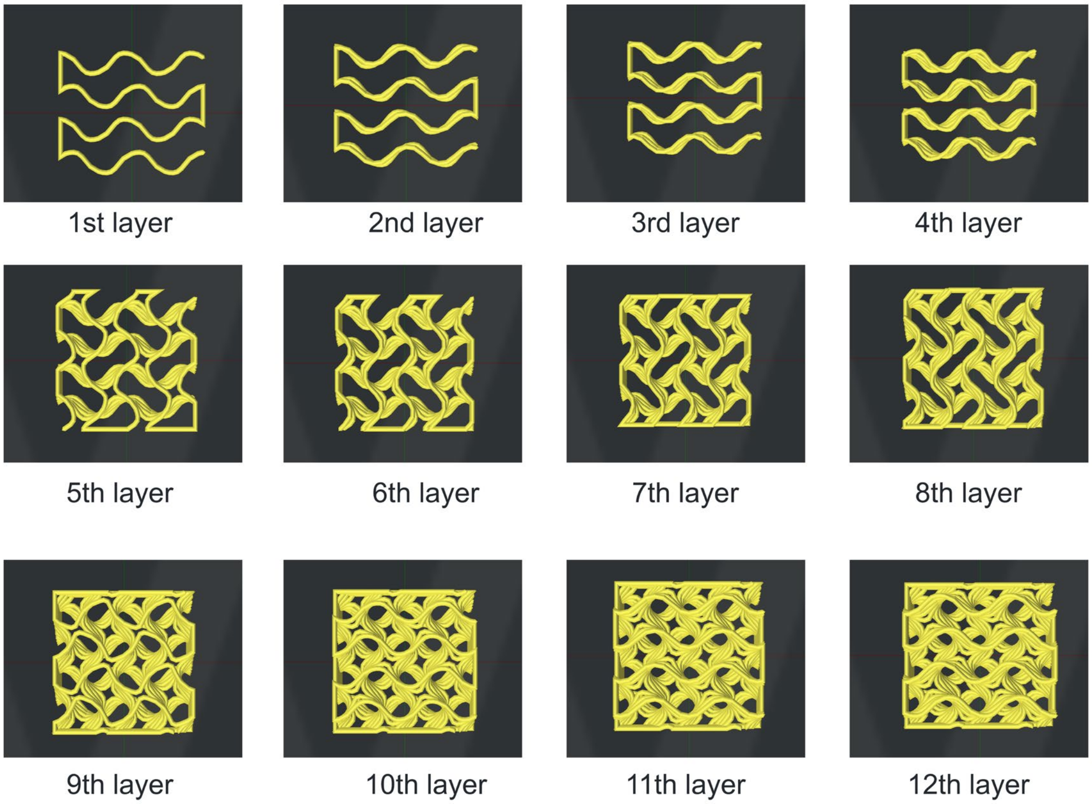

Supplementary Fig. 10. FDM-printed layer-by-layer paths of Gyroid.

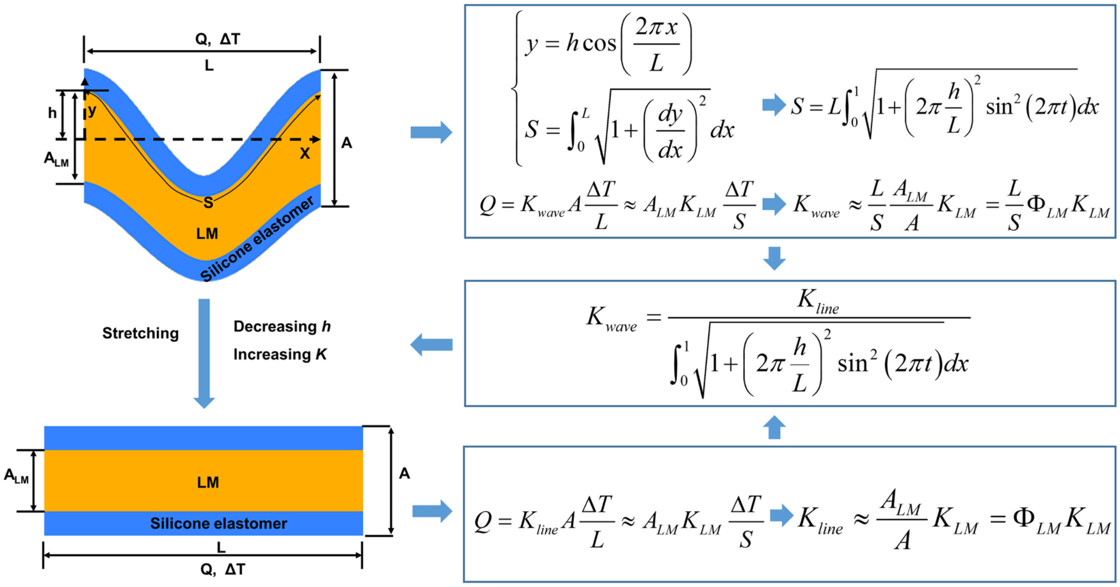

Supplementary Fig. 11. The simplified theoretical model of stretching-enhanced thermal conductivity in the stretching direction for LMSE. The wave-shaped segment of the Gyoid-type LM skeleton is straightened along the stretching direction. For the segment length of L and cross-section area of A (including the LM skeleton cross-section area of A_{LM}, indicating the volume fraction of LM with $\Phi_{LM}=A_{\mathrm{LM}}/A$), the relation between the thermal flux (Q) and the temperature difference (ΔT) can be used to estimate the effective thermal conductivity (K) of LMSE. To simplify the theoretical analysis, we assume that the thermal conduction of LMSE with a large content of the LM (Φ_{LM}>40%) is mainly determined by the LM skeleton due to the low heat conductivity of the silicone matrix (K_{MA}=0.2 W/mK) compared with LM (K_{LM}=33.4 W/mK for gallium). In addition, the volume fraction of LM is kept the same for the two cases (before and after stretching). It is noteworthy that the thermal conduction path length of the wave-shaped LM skeleton is the curve length of S for the segment length of L. The simplified theoretical model indicates that increasing the bending (corresponding to wave amplitude of h) of the LM skeleton would extend the thermal path and decrease the effective thermal conductivity of K_{wave}. Thus, the wave-shaped segment of the LM skeleton is straightened along the stretching direction, leading to a decrease in h and an increase in K_{wave}. It is noteworthy that the upper limit value of K_{wave} is K_{line} (corresponding to h=0). The theoretical model is consistent with the numerical and experiment results. For example, K_{wave}=27.1 W/mK (for Φ_{LM}=81.4%) approaches to $K_{line}= \Phi_{LM}K_{LM}+(1-\Phi_{LM})K_{MA}$= 27.2W/mK under the strain of 300% ($h\approx 0$).

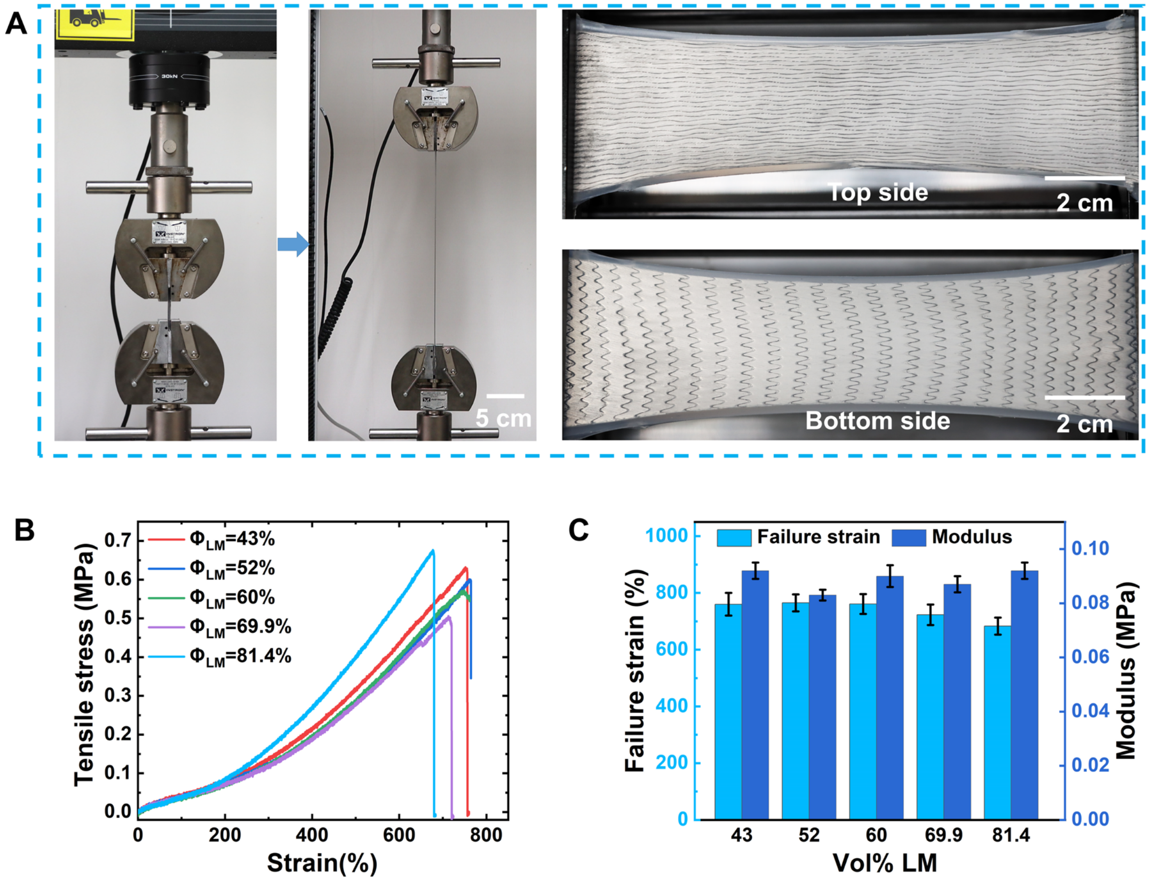

Supplementary Fig. 12. Mechanical characteristics of LMSE. (A) The test platform and photos of the LMSE with Φ_{LM}=81.4% when strained to 600%. **(B)** Curves of strain-stress for the LMSE versus the LM volume ratio. **(C)** Modulus and failure strain versus the LM volume ratio. Values in **C** represent the mean with error bars (n = 3; independent samples).

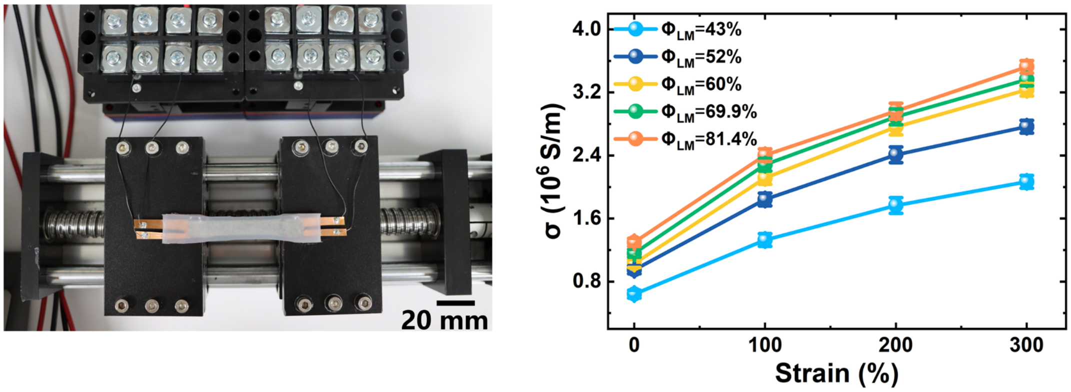

Supplementary Fig. 13. The test platform and volumetric electrical conductivity of LMSE versus the strain. Values represent the mean with error bars (n = 3; independent samples).

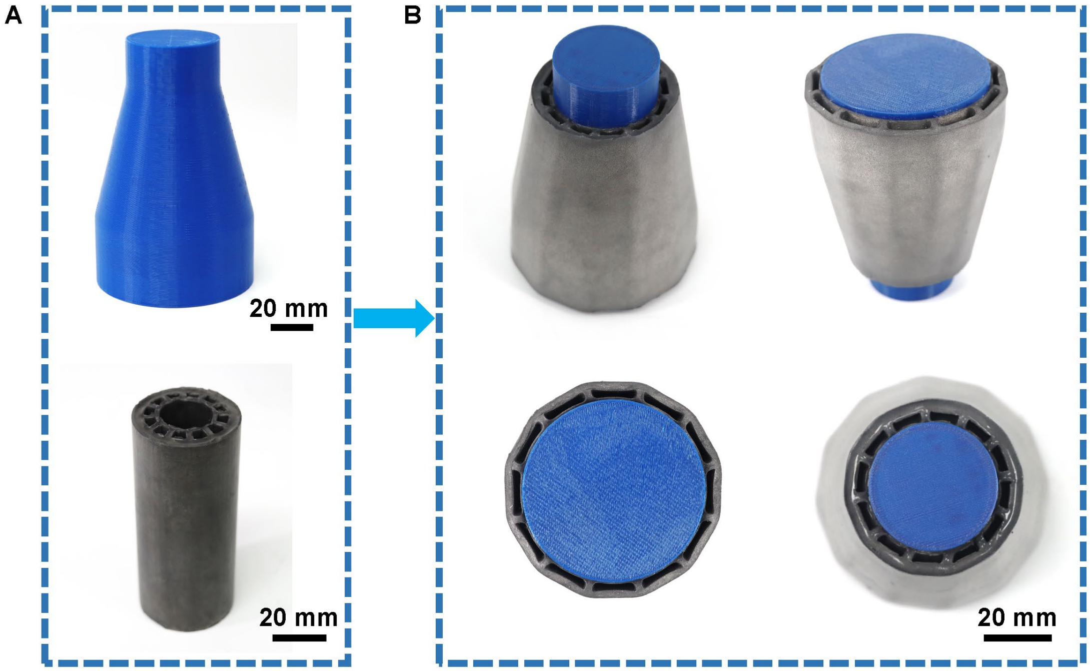

Supplementary Fig. 14. Photographs of the sleeve-shaped LMS-ACE conformal to the even cone body with the diameter of the upper end D_1=40 mm, and the diameter of the lower end D_2=70 mm.

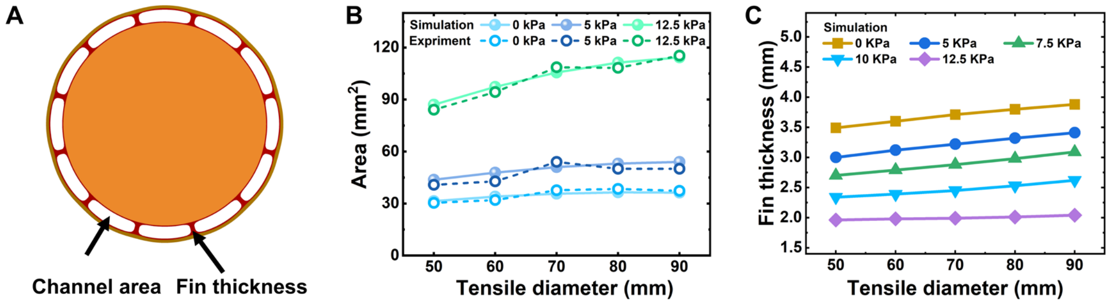

Supplementary Fig. 15. Experiment and simulation results of the sleeve-shaped LMS-ACE conformal to the cylindrical surface with different diameters. **(A)** The diagram of channel area and fin thickness. **(B)** The curves of LMS-ACE cross-section area versus tensile diameter. **(C)** The curves of LMS-ACE fin thickness versus tensile diameter.

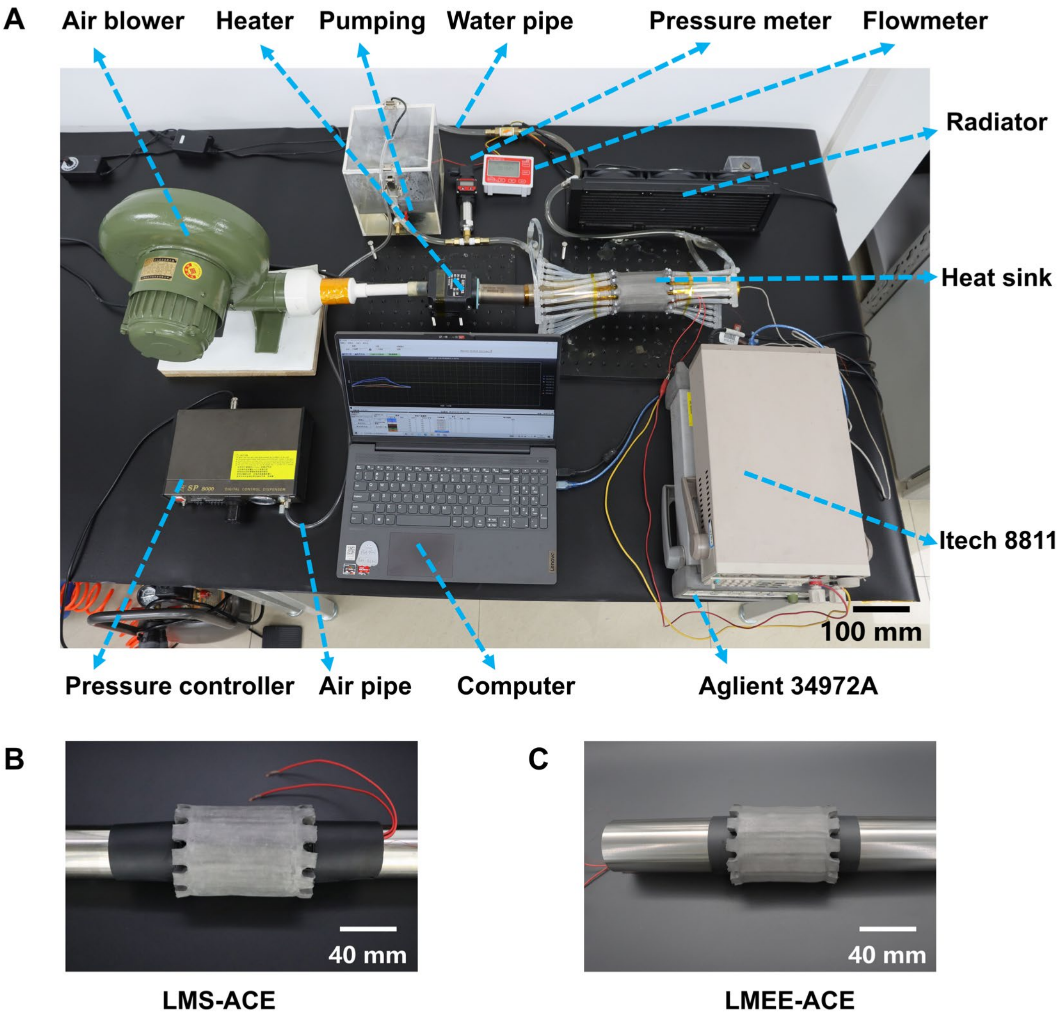

Supplementary Fig. 16. (A) The test platform of waste heat harvesting by the combination of FTED with **(B)** LMS-ACE and **(C)** LMEE-ACE. There are 4 FTEDs on the exhaust pipe. The single FTED has 168 pairs of P/N thermoelectric legs with a size of 1.3 mm × 1.3 mm × 3 mm, and a total area of 27.8 mm × 66.2 mm.

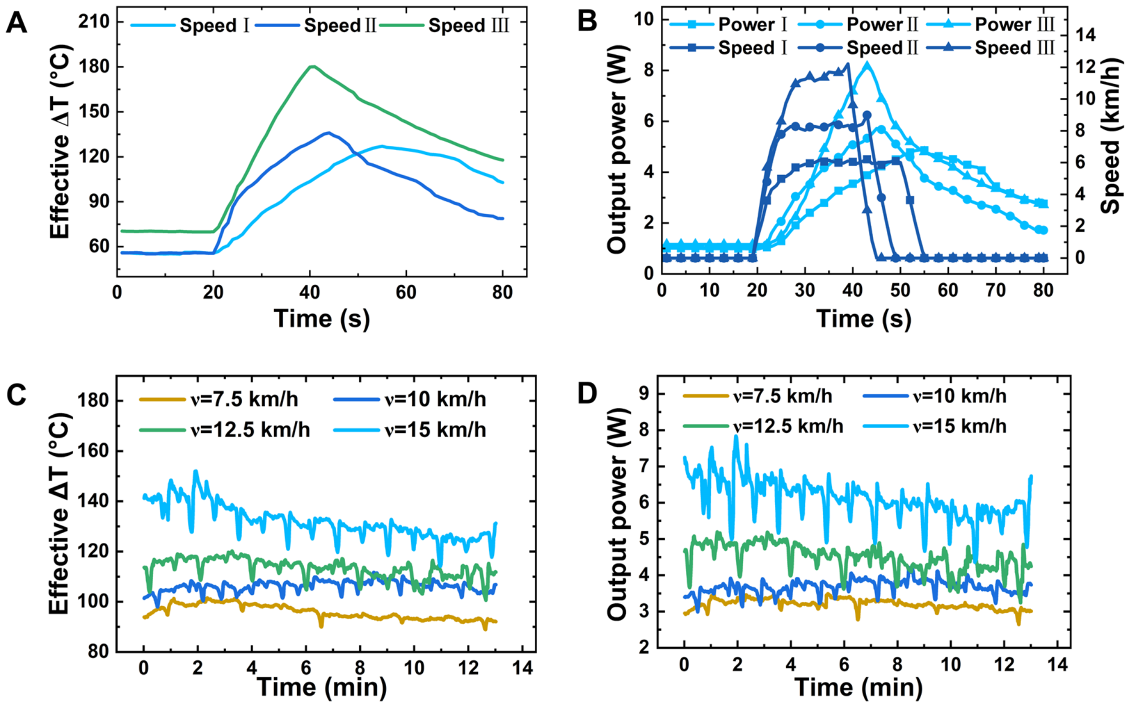

Supplementary Fig. 17. The performance of the FTED with the different workloads of the tractor. **(A)** The effective exhaust temperature in the tractor climbing condition with different speeds determining **(B)** the output power. **(D)** The effective exhaust temperature in the tractor flat driving condition with different speeds determining **(C)** the output power.

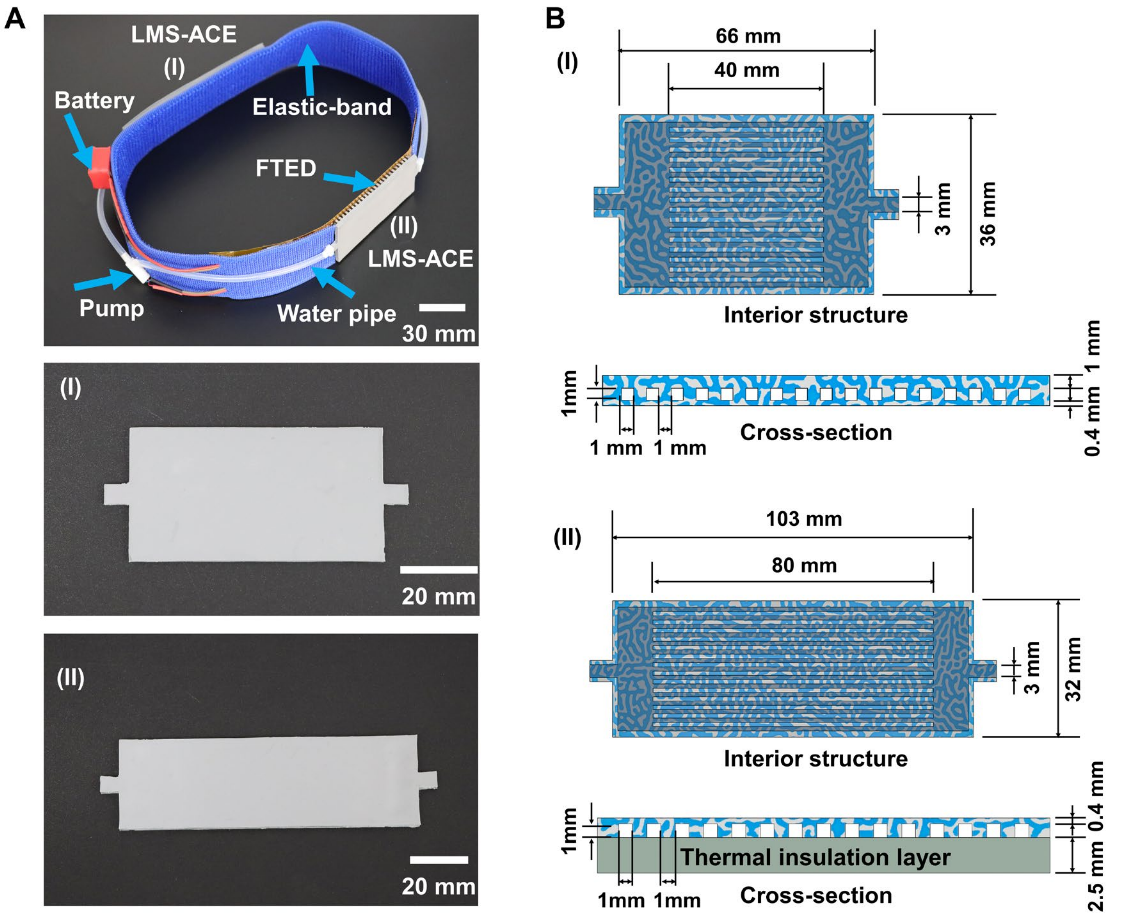

Supplementary Fig. 18. The characterization of the wearable smart cooling headband. **(A)** Photograph of the wearable cooling headband. **(B)** The characterization of the two LMS-ACEs' interior structures and cross-sections.

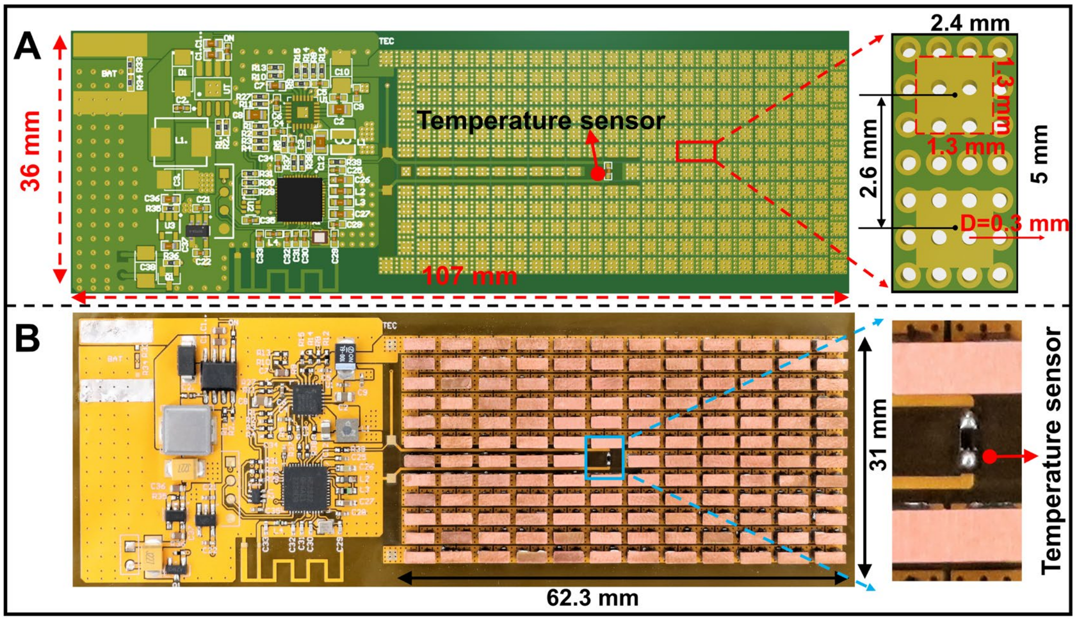

Supplementary Fig. 19. The intelligent cooling device. **(A)** The electronic component layout of the FPCB diagram. **(B)** Photograph of the intelligent cooling device.

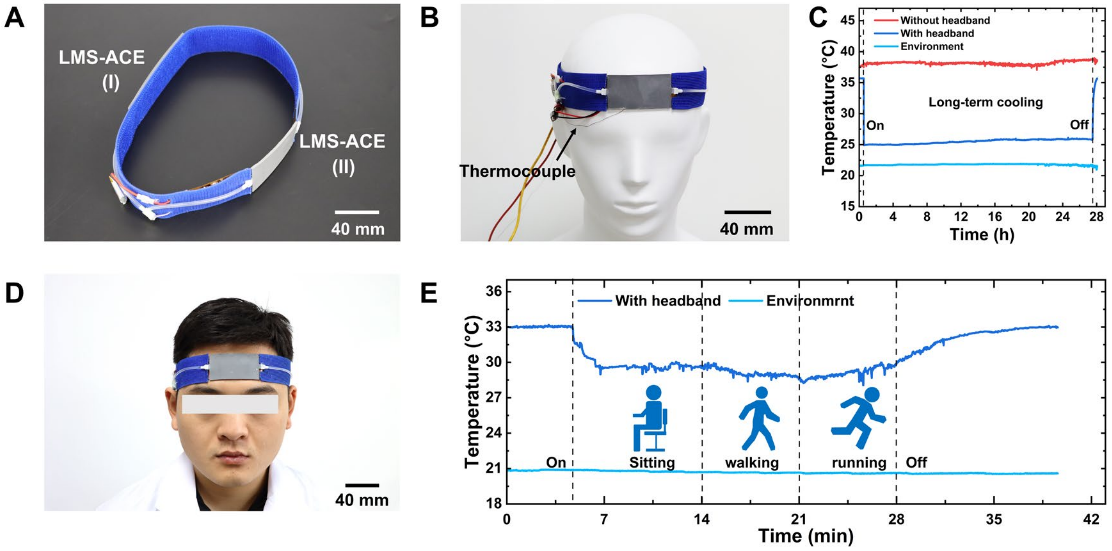

Supplementary Fig. 20. Performance of the wearable cooling headband with only LMS-ACE without FTED. (A) Photograph of the wearable cooling headband. **(B)** Photograph of the wearable cooling headband worn on mannequin. The heating film (with the size of 30 mm × 60 mm, input voltage 1.72 V, input current 0.6 A, input power 1.032 W, corresponding to the thermal power density of 573.3 W/m^2) was attached to the mannequin head. The cooling headband was attached to the heating film, and a thermocouple was placed between the heating film and the cooling headband to obtain real-time temperature data. **(C)** Long-term operation test of the headband cooling performance (environment temperature 21.5 ℃). Without the cooling headband, the model's temperature reached 38.1 ℃. After wearing the cooling headband, the model's head temperature was reduced from 35 °C to 24.9 °C. This cooling effect could be maintained for a long-term operation (>27 hours). **(D)** Optical images of the cooling headband worn on the head. **(E)** The cooling performance of headband wearing on the human head. The temperature of the human forehead is 33.0 °C without cooling headband. The cooling headband could maintain the forehead temperature between 28 °C and 29 °C under different conditions of sitting, walking, and running.

Supplementary Note 1

Simulating the mechanical deformation of the LMS-ACE. The 3D FEA model for the LMS-ACE utilized a commercial software package (Ansys 19.2). The static structure modules were employed to solve mechanical deformations. The LMS-ACE was simulated by calculating total deformation and contact pressure on different rigid bodies. The mechanical properties of materials used in the model are summarized in **Supplementary Table 1** and **Supplementary Table 2**. Since the LMS-ACE consisted of the LMS and an elastomer, which is a hyperelastic material, it had an inner diameter of 21 mm and an outer diameter of 45 mm with a total of 12 channels. The mechanical properties of the LMS-ACE were determined through uniaxial tensile tests and fitted with ANSYS Engineering Data, representing a simplified homogeneous model. As an incompressible hyperelastic material, the stress-strain relationship exhibited high nonlinearity; hence, the Yeoh 3rd Order constitutive model was selected to accurately capture deformation behavior. Rigid bodies were constructed using a high elastic modulus material steel while being constrained by displacement conditions to ensure minimal deformation. A frictional method was employed as the contact type between the LMS-ACE and rigid body surfaces, utilizing the Augmented Lagrange algorithm.

Supplementary Table 1. Value of the Yeoh 3rd Order model for LMS-ACE

Material	*Density* (kg/m^3)	*C10* (Pa)	*C20* (Pa)	*C30* (Pa)	*D1* (Pa^{-1})	*D2* (Pa^{-1})	*D3* (Pa^{-1})
LMS-ACE	3621.3	13887	430.58	-2.5589	0	0	0

Supplementary Table 2. Value of the mechanical parameter for rigid body

Material	*Density* (kg/m^3)	*Young's Modulus* (GPa)	*Poisson Ratio*
Structure steel	7850	200	0.3

The comprehensive simulation encompassed four phases to characterize the deformation. 1) Radial displacements were applied to expand the inner surface of the LMS-ACE. 2) The rigid body was relocated to the axial center of the expanded LMS-ACE and constrained by displacement. 3) The constraints on LMS-ACE were released, allowing it to freely contract towards the rigid body. 4) Surface pressures were exerted on each channel of the LMS-ACE. This entire process restricted axial movement of the LMS-ACE. The Yeoh 3rd Order constitutive model was employed.

$$W=\sum_{i=1}^{3}c_{i0}\left(\overline{I}_1-3\right)^i+\sum_{k=1}^{3}\frac{1}{D_k}(J-1)^{2k} \tag{1}$$

where, W was the strain energy density， c_{i0}(i=1,2,3) was the material constant parameter, D_k(k=1,2,3) was incompressibility parameter. $\overline{I_1}$ was the first strain invariant. J was the volume ratio of deformation before to after. In this study, the LMS-ACE was set to be incompressible, that was, J=1.

Simulating the mechanical deformation and thermal conductivity of LMSE. The mechanical deformations and heat transfer were effectively obtained by modules of the static structure and Fluent. The LMSE was modeled by calculating the total deformation and thermal conductivity while considering the mechanical and thermal properties of the materials used in the model (as summarized in **Supplementary Table 1** and **Supplementary Table 3**). The LMSE consisted of both the LM and elastomer, forming a cubic model. The material properties of this composite were equivalent to those of LMS-ACE, which can be considered as a homogeneous hyperelastic material. It is important to note that the volume of the LM remained constant throughout. The specific boundary conditions were applied for deformation simulation: 1) The displacement stretch was constrained along two surfaces in one direction while allowing free deformation in other directions; 2) Rotation displacement on four surfaces (excluding stretched surfaces) was constrained to zero. Post-processing allowed the deformation values for both LM and elastomer models to be obtained. In order to determine the thermal conductivity of LMSE, heat transfer processes within the models were simulated. A fixed temperature difference (100 k) between front/back surfaces along X, Y, Z directions was set while isolating other surfaces. Subsequently, CFD-post processing enabled calculation of total heat Q on heat source surface using Fourier's law equation to obtain equivalent thermal conductivity.

$$\lambda = \frac{Q \cdot d}{\Delta T \cdot A} \tag{2}$$

where, ΔT is the absolute temperature difference between the two ends of the intercepted model, Q is the heat flux of the heat source surface, A is the heat source surface area of the model, d is the length of the intercepted model.

Supplementary Table 3. Thermal parameters used in the finite element simulations

Material	*Thermal conductivity* (W/mK)	*Density* (kg/m^3)	C_p (J/kg·K)
Pure silicone	0.2	1060	1700
Ga	33.4	5904	371
Water	0.613	1000	4179
