# Supplementary material for: Hydraulic-driven adaptable morphing active-cooling elastomer with bioinspired bicontinuous phases
Source: Nat Commun. 2024 Feb 8;15:1179. doi: 10.1038/s41467-024-45562-y (PMC10853206; doi:10.1038/s41467-024-45562-y)
Supplement: Supplementary file 3 — Description of Additional Supplementary Files [file 41467_2024_45562_MOESM3_ESM.pdf]

Description of Additional Supplementary Files

Supplementary Movie 1: Presentation of the LMS-ACE fabrication process.

Supplementary Movie 2: Demonstration of the soft gripper with hydraulic-driven conjoint functions of actuation and cooling.

Supplementary Movie 3: Layer-by-layer printing paths for different micropatterns.

Supplementary Movie 4: The topological microstructures of LMS embedded in the silicone elastomer under large stretching.

Supplementary Movie 5: The 3D micro X-ray computed tomography of the Gyroid-type LMS microstructure of LMS.

Supplementary Movie 6: Active thermo-fluidic hydraulic-driven adaptation of the LMS-ACE to the concave shape.

Supplementary Movie 7: Performance test of the FTED combined with LMS-ACE under conditions of tractor farming operations.

Supplementary Movie 8: The composition and working principle of the wearable smart cooling headband.
